# Supplementary material for: Comparison of Physicochemical Properties of Noodles Fortified with Commercial Calcium Salts versus Calcium Citrate from Oyster Shells
Source: Foods. 2023 Jul 13;12(14):2696. doi: 10.3390/foods12142696 (PMC10379373; doi:10.3390/foods12142696)
Supplement: Supplementary file 1 [file foods-12-02696-s001.zip › foods-2473859-supplementary.pdf]

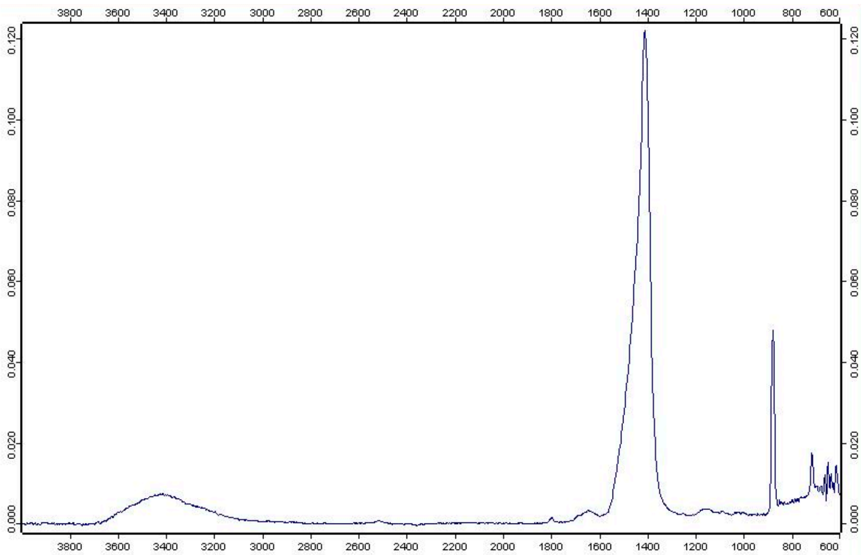

Supplementary Figure S1 IR spectra of oyster shell powders.

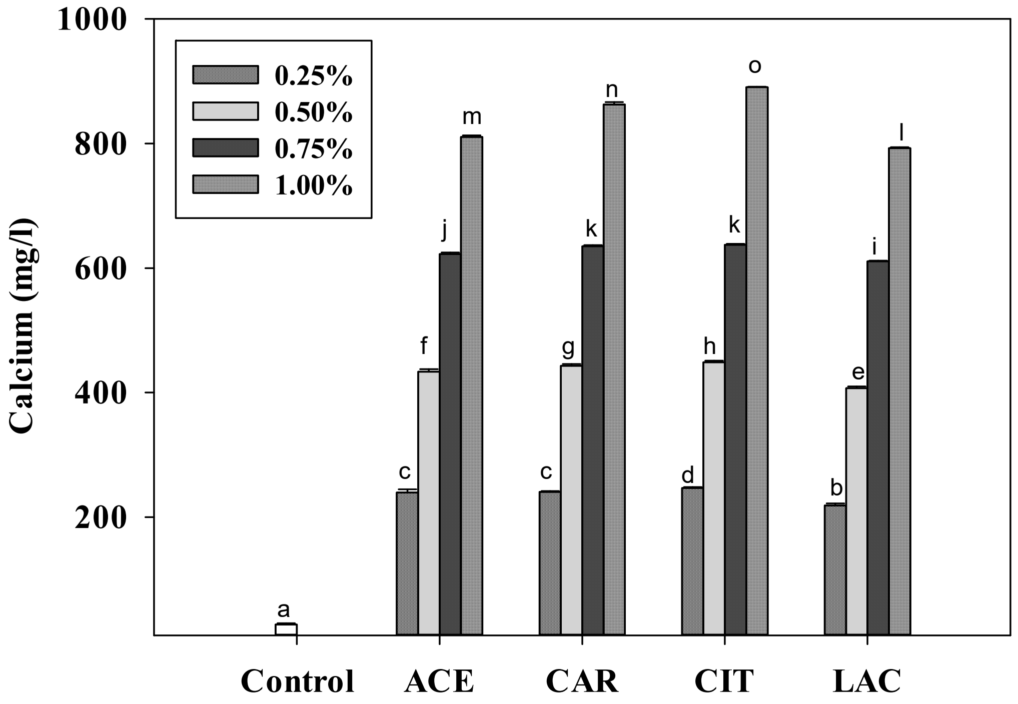

Supplementary Figure S2 Effect of kinds and amounts of calcium salts on the calcium content of drying noodles.

ACE, Calcium acetate; CAR, Calcium carbonate; CIT, Calcium citrate; LAC, Calcium lactate.
